# Combined *in vitro* IL-12 and IL-15 stimulation promotes cellular immune response in dogs with visceral leishmaniasis

**DOI:** 10.1371/journal.pntd.0008021

**Published:** 2020-01-21

**Authors:** Sidnei Ferro Costa, Vinícius Oliveira Gomes, Marilene Oliveira dos Santos Maciel, Larissa Martins Melo, Gabriela Lovizutto Venturin, Jaqueline Poleto Bragato, Gabriela Torres Rebech, Catiule de Oliveira Santos, Bárbara Maria Nascimento de Oliveira, Geraldo Gileno de Sá Oliveira, Valéria Marçal Felix de Lima

**Affiliations:** 1 Department of Animal Clinic, Surgery and Animal Reproduction, São Paulo State University (UNESP), School of Veterinary Medicine, Araçatuba, Brazil; 2 Oswaldo Cruz Foundation, Gonçalo Moniz Research Center, Laboratory of Structural and Molecular Pathology (LAPEM), Rua Waldemar Falcão, Candeal, Salvador, Bahia, Brazil; University of Iowa, UNITED STATES

## Abstract

Domestic dogs are the main reservoir of *Leishmania infantum*, a causative agent of visceral leishmaniasis (VL). The number of human disease cases is associated with the rate of canine infection. Currently available drugs are not efficient at treating canine leishmaniasis (CanL) and months after the treatment most dogs show disease relapse, therefore the development of new drugs or new therapeutic strategies should be sought. In CanL, dogs lack the ability to mount a specific cellular immune response suitable for combating the parasite and manipulation of cytokine signaling pathway has the potential to form part of effective immunotherapeutic methods. In this study, recombinant canine cytokines (rcaIL-12, rcaIL-2, rcaIL-15 and rcaIL-7) and soluble receptor IL-10R1 (rcasIL-10R1), with antagonistic activity, were evaluated for the first time in combination (rcaIL-12/rcaIL-2, rcaIL-12/rcaIL-15, rcaIL-12/rcasIL-10R1, rcaIL-15/rcaIL-7) or alone (rcasIL-10R1) to evaluate their immunomodulatory capacity in peripheral blood mononuclear cells (PBMCs) from dogs with leishmaniasis. All the combinations of recombinant proteins tested were shown to improve lymphoproliferative response. Further, the combinations rcaIL-12/rcaIL-2 and rcaIL-12/rcaIL-15 promoted a decrease in programmed cell death protein 1 (PD-1) expression in lymphocytes. These same combinations of cytokines and rcaIL-12/rcasIL-10R1 induced IFN-γ and TNF-α production in PBMCs. Furthermore, the combination IL-12/IL-15 led to an increased in T-bet expression in lymphocytes. These findings are encouraging and indicate the use of rcaIL-12 and rcaIL-15 in future *in vivo* studies aimed at achieving polarization of cellular immune responses in dogs with leishmaniasis, which may contribute to the development of an effective treatment against CanL.

## Author summary

Dogs are the main reservoir of *Leishmania infantum*, a protozoan parasite that causes lethal systemic disease in human beings (Visceral Leishmaniasis, VL) and dogs (Canine Leishmaniasis, CanL). In dogs, the disease is often associated with extensive skin lesions. Curbing canine infection could help control human VL. Currently available drugs are not effective in treating CanL, and treated dogs frequently relapse, even when removed from an endemic area. Developing new drugs or alternative therapeutic strategies are essential for the proper treatment of CanL. Dogs that acquire the parasite can develop the disease or control the infection, depending on the body’s immune response. Cellular immune responses lead to infection control and prevent the disease from developing. Some proteins called cytokines, produced mainly by cells of the immune system, can favor the development of cellular immune responses. In this study, several recombinant cytokines and/or one cytokine blocking protein were tested, alone or in combination, for their ability to promote cellular immune response in dogs with leishmaniasis. One of the combinations of recombinant protein tested was the best at facilitating this cellular immune response. The findings reported here are encouraging and indicate the use of a combination of two recombinant cytokines (rcaIL-12/rcaIL-15) in future immunotherapeutic trials for CanL.

## Introduction

The zoonotic form of visceral leishmaniasis (VL) is caused by the obligate intracellular protozoan *Leishmania infantum* (syn. *L*. *chagasi*, in Americas) [1,2]. VL is the most severe form of leishmaniasis and is fatal in 95% of untreated cases [3]. VL is distributed worldwide, occurring mainly in tropical and subtropical regions with approximately 300.000 new infections each year and an estimated 20.000 to 50.000 deaths [4]. Domestic dogs are considered the main reservoir of the parasite in urban areas [5]. In endemic areas, there is a correlation between the prevalence of seropositive dogs and number of human cases of VL [6–8], suggesting that controlling infection and/or disease in dogs (CanL) could contribute to effectively curbing human disease [8].

The current treatments available for CanL have leishmanicidal and leishmaniostatic effects [9] and lead to a reduction in parasite load, infectiousness, and resolution of clinical signs [10]. However, most dogs remain infected and experience disease relapse months after treatment withdrawal, once again becoming a source of parasites for other healthy dogs and human beings [10]. The frequent disease relapses following currently available therapy suggests that new drugs or therapeutic approaches for CanL, such as the association of existing drugs with immunostimulants, should be sought [11].

Untreated asymptomatic dogs (generally resistant to infection by *L*. *infantum*) develop an efficient cellular immune response (Th1) with simultaneous production of IFN-γ, IL-2, and IL-12 [12–14], and an activation of leishmanicidal mechanisms in infected macrophages [15,16]. In contrast, symptomatic dogs (susceptible to the infection) mount an exacerbated humoral immune response (Th2) that may be accompanied by increased production in of IL-10 [17]. In addition, susceptible dogs present increased expression of programmed cell death 1 (PD-1) and PD-1 ligands (PD-L1 and PDL-2), in splenic cells [18]. Such heightened expression of PD-1 and PD-1 ligands may suppress lymphoproliferation and alters the production of Th1 cytokines, contributing to the development of the disease [19]. Manipulations of certain cytokine signaling pathways may favor control over the parasite in infected individuals [12,13,17,20–23]. Interestingly, most human beings who mount inappropriate adaptive immune responses for combating *L*. *infantum* and develop the disease, subsequent to treatment with pentavalent antimonials or amphotericin B, reprogram their specific immune responses [21,22], maintain the parasite replication under control and show no disease recurrence.

Human patients with VL lack the ability to mount lymphoproliferative response and IFN-γ production following peripheral blood mononuclear cells (PBMCs) *in vitro* stimulation with soluble *Leishmania* antigens (SLA), that would relate to development of the disease [21]. However, when PBMCs from such patients are stimulated with SLA in combination with recombinant human interferon gamma (rhuIFN-γ) and interleukin-2 (rhIL-2) they present restoration of lymphoproliferative response [21]. Further, stimulation of PBMCs with SLA together with rhuIL-12 or blocking signaling with anti-IL-10 antibodies results in both restoration of lymphoproliferative response and production of IFN-γ [21,22]. In naturally *L*. *infantum*-infected sick dogs, treatment of PBMCs with rcaIL-12 generates an increase in IFN-y mRNA expression or protein production [20,24] and a tendency to enhance lymphoproliferative response to SLA [20]. To our knowledge, studies evaluating the activities of IL-7 in humans or dogs with visceral leishmaniasis have not yet been conducted. Although interfering with a single cytokine pathway, with agonistic or antagonistic molecules, can drive responses in cells of the immune system, simultaneous intervention in two or more cytokines signaling pathways may elicit stronger responses in these cells, even in settings with low cytokine concentrations [25–28]. Successful attempts to modify immune responses in human or dog PBMCs using a combination of cytokines showing additive or synergistic effects have already been performed. For instance, rhuIL-15 combined with rhuIL-12 promotes higher levels of IFN-γ, compared with rhuIL-15 alone or rhuIL-12 alone, and may generate effective responses to infections caused by intracellular parasites [29]. In addition, rcaIL-12 and rcaIL-2 together stimulate efficient production of IFN-γ, whereas rcaIL-12 or rcaIL-2 alone do not induce this effect or does so in limited amounts [30,31].

In this study, canine recombinant proteins (rcaIL-12, rcaIL-2, rcaIL-15 or rcaIL-7, rcasIL-10R1 with antagonistic activity) were evaluated for their capacity to reprogram responses in PBMCs from dogs with leishmaniasis. These recombinant proteins were assessed in combination or alone. The responses studied were lymphoproliferation, the presence of PD-1 on lymphocyte surface, the production of IFN-γ, TNF-α, IL-10, and NO_2_, and the synthesis of T-bet and GATA3 in lymphocytes. Recombinant proteins potentially capable of stimulating macrophages to control replication or destroy *L*. *infantum* could have a positive impact on the development of immunotherapeutic protocols for CanL.

## Methods

### Animal screening and sample collection

This study was approved by the Brazilian Society of Science on Laboratory Animals/Brazilian College of Animal Experimentation (SBCAL/COBEA), and received approval from the Institutional Committee for Animal Care and Use (São Paulo State University (UNESP), Araçatuba, School of Veterinary Medicine (FMVA), under protocol no. 00765–2017. The license approved covered the use of healthy negative control and diseased dogs.

Five healthy dogs from Araçatuba, São Paulo, with negative results for the detection of *Leishmania* DNA by real-time PCR, as well as complete blood counts and mean serum biochemistry parameters within reference ranges, were used as negative controls. These dogs were pet animals and their owners gave written permission for the experiment procedures. Ten dogs were selected from the Araçatuba Zoonosis Control Center that showed at least three of the following clinical signs of CanL: onychogryphosis, cachexia, ear-tip injuries, periocular lesions, alopecia, skin lesions or lymphadenopathy (see supplementary material, S1 Table).

Blood samples from both groups, healthy controls and diseased dogs, were collected in tubes without EDTA to obtain serum for the evaluation of biochemical profiles (S2 Table) and to perform indirect ELISA (S1 Table) for the detection of anti-*Leishmania* antibodies [32]. Blood was also collected in EDTA tubes for complete blood counts (CBC) (S3 and S4 Tables) and PBMCs isolation. Real-time PCR for the detection of *Leishmania* DNA was performed in canine blood samples using a calibration curve obtained from the DNA of 10^2^ to 10^7^ *Leishmania* promastigotes, as previously described [33]. Sera samples were also tested for *Dirofilaria immitis* antigens and antibodies reactive to *Anaplasma phagocytophilum*/*Anaplasma platys*, *Ehrlichia c*anis/*Ehrlichia ewingii* and *Borrelia burgdorferi* using the SNAP 4Dx Plus rapid test (IDEXX Laboratories, Inc. USA), in accordance with the manufacturer recommendations. In addition, blood samples were tested for *Ehrlichia spp* DNA by conventional PCR using a slightly modified protocol previously described by Labruna et al. 2007 [34].

### Isolation of peripheral blood mononuclear cells

PBMCs from healthy controls and diseased dogs were isolated by gradient centrifugation using Histopaque 1077 (Sigma, USA), according to the manufacturer’s recommendations. Isolated cells were then washed three times in phosphate buffered saline (PBS, pH 7.2) and suspended in RPMI 1640 (Sigma, USA) supplemented with inactivated 10% fetal bovine serum (FBS) (Gibco, USA), 0.03% L-glutamine (Sigma, USA), 100 IU/mL penicillin (Sigma, USA) and 100 mg/mL streptomycin (Sigma, USA).

### GeneBank data

Data from GenBank accession numbers DQ845341, XM_844053, and XM_005620306.1 were used to design DNA constructs to produce rcaIL-7, rcaIL-15, and rcasIL-10R1. To evaluate similarities between human and canine T-bet and GATA3 proteins, GenBank accession numbers NP_037483, CAA38877, XP_548164, and XP_005617214) T-bet and GATA3 proteins were used.

### Production of canine recombinant cytokines and soluble IL-10R1 receptor

Recombinant canine IL-12 was produced using a baculovirus-insect cell system, as previously described [31]. Recombinant canine IL-2, rcaIL-7 and rcasIL-10R1 (soluble recombinant extracytoplasmic domain of the IL-10 receptor α chain) were also obtained using a baculovirus-insect cell system. For this, DNA constructs were designed to encode in tandem a signal peptide, either mature IL-2 [35], mature IL-7 (GeneBank, accession DQ845341) or casIL-10R1 (GeneBank, accession XM_005620306.1), followed by a spacer (only in IL-2 and IL-7 constructs) and His-tag. DNA encoding the signal peptide from *Autographa californica* multiple nucleopolyhedrovirus (AcMNPV) glycoprotein 64 kDa (GP64) [36] was used in the constructs. All DNA constructs were synthetized by GenScript using codons optimized for translation in *Trichoplusia ni* (Piscataway, USA). Bacmids (AcBacΔCC-GP64-IL-2E6H, AcBacΔCC-GP64-IL-7E6H, and AcBacΔCC-GP64-sIL-10R16H) were generated and purified from the DH10BacΔCC *Escherichia coli* strain [31]. The human IL-10R1 extracytoplasmic domain (GeneBank, accession NP_001549) was used as a model to identify the casIL-10R1 polypeptide chain using a freely available online tool (https://tmdas.bioinfo.se/DAS/index.html). Viral stocks (AcBacΔCC-GP64-IL-2E6H, AcBacΔCC-GP64-IL-7E6H and AcBacΔCC-GP64-sIL-10R16H) were titrated using an end-point method in Sf-9 cells (Invitrogen, Carlsbad, USA), and recombinant protein production and purification was performed, as previously described [31]. Recombinant canine IL-15 was generated in the BL21(DE3)pLysS *E*. *coli* strain (Invitrogen) transformed by a plasmid DNA construct (*pRSET-mcaIL-15-opt-3S*), according to the manufacturer’s instructions, and subsequently purified by affinity chromatography [30], then refolded in 100 mM Tris-HCl, 500 mM glycine, 1 mM oxidized glutathione and 10 mM reduced glutathione (pH 8.0). To produce the protein, a DNA construct was synthetized encoding mature canine IL-15 (Genebank, accession XM_844053) using codons optimized for translation in *E*. *coli* (GenScript). In addition, endotoxin concentrations from all purified recombinant proteins were determined using Limulus Amebocyte Lysate (Gel-clot Method, Pyrotell, USA) [37], which results in low levels of endotoxins (<0.03 EU of endotoxin per mg of protein). All purified recombinant proteins were confirmed by Western blot assays using anti-his antibodies. Biological activity was verified as follows: both rcaIL-2 and rcaIL-15 were shown to induce CTLL-2 cell proliferation [31]; RcaIL-12 in combination with rcaIL-2 promoted IFN-γ production in canine PBMCs [30]; rcaIL-7 and rcaIL-15 both stimulated the proliferation of canine PBMCs. Finally, rcasIL-10R1 was also shown to inhibit the proliferation of canine IL-10-treated MC/9 murine mast cells (ATCC CRL 8306) [38].

### Lymphoproliferation assay

To assess the proliferation of lymphocytes, PBMCs were stained with carboxyfluorescein diacetate succinimidyl ester (2.5 μM) (CFSE, CellTrace, Invitrogen, UK) for 10 min at 37°C, according to the manufacturer’s recommendations and a previously published protocol [39]. Stained PBMCs were cultured in sterile 96-well plates (1×10^6^/mL) in RPMI 1640 medium (Sigma, USA), either alone (negative control) or with the following recombinant canine proteins: rcaIL-2 (2 ng/mL), rcaIL-7 (40 ng/mL), rcaIL-12 (20 ng/mL), rcaIL-15 (20 ng/mL), rcasIL-10R1 (4 μg/mL). These recombinant proteins were tested in the following combinations: rcaIL-12/rcaIL-2, rcaIL-12/rcaIL-15, rcaIL-12/rcasIL-10R1, rcaIL-7/rcaIL-15, or rcasIL-10R1 alone. Cells were cultured either in the presence or absence of 20 μg/mL of SLA (MHOM/BR/00/MERO2), as previously described [40]. Cultures containing the mitogen phytohemagglutinin-M (PHA-M, 5 μL/mL) were used as positive controls. CFSE-unmarked PBMCs were used to verify CFSE staining. PBMCs were cultured for 5 days at 37°C under 5% CO_2_. Ten thousand events were acquired in a flow cytometer (BD C5 Accuri Flow Cytometer, USA) and data analysis was performed using BD Accuri C6 software, version 1.0 (BD Biosciences, CA, USA). Cell populations with similar size and complexity to the lymphocyte population were gated and evaluated by positive CFSE labeling (S1 Fig). Assays were repeated in duplicate under identical conditions without CFSE staining. Cell culture supernatants were used to determine IFN-y, TNF-α and IL-10 concentrations by capture ELISA (R&D Systems, USA), according to the manufacturer’s recommendations and NO_2_ determination by Greiss reagent. Cells were also used to determine the protein expression of PD-1, T-bet and GATA3 transcription factors by flow cytometry (described below).

### Flow cytometry analysis for labeling PD-1, T-bet and GATA3 in PBMCs

To detect PD-1 expression, PBMCs were suspended in PBS containing 1% bovine serum albumin, 0.1% sodium azide and 20% fetal bovine serum, to block the Fc receptor (FcR). Cells were then mixed with either the PE-conjugated monoclonal anti-Human CD279 (PD-1) antibody [18,19] or an isotype control (BD Pharmigen, USA), according to the manufacturer instructions. Ten thousand events were acquired on the FL2 channel of a flow cytometer, and data analysis was performed as described above in the lymphoproliferation assay section (S2 Fig).

To evaluate T-bet and GATA3 expression, PBMCs were fixed and permeabilized with a commercial buffer (eBioscience Bioscience, USA), according to the manufacturer's instructions. Cells were mixed with the FITC-conjugated anti-human monoclonal antibody T-bet (R&D Systems) and with the PE-conjugated anti-human monoclonal GATA3 (R&D Systems), or control isotypes (R&D Systems), according to the manufacturer's instructions. The similarity between human (GenBank, accession # NP_037483 and CAA38877) and canine (XP_548164 and XP_005617214) T-bet and GATA3 proteins is 93 and 96% respectively. Ten thousand events were acquired on channels FL1 and FL2, and cytometric analysis was performed as described above in the lymphoproliferation assay section (S3 Fig).

### NO_2_ determination

As a surrogate marker of NO_2_, the Griess method was used to determine nitrite concentrations in supernatants of PBMCs cultured for 5 days with or without the addition of SLA and/or combinations of recombinant proteins [41]. For this, 50 μL of culture supernatant was added to 50 μL of Griess reagent (one part 0.1% NED and one part 1% sulfanilamide in 5% phosphoric acid). After 5 min of incubation at room temperature, optical density readings were taken at 540nm using a 96-well plate reader (Spectra Count, Packard Bio Science Company, Meriden, CT, USA). Nitrite concentrations in cell culture supernatants were determined using a standard sodium nitrite curve (range: 3–200 μM).

### Statistical analysis

Statistical analysis was performed using GraphPad Prism v6 software (GraphPad Software, Inc., La Jolla, CA, USA). All statistical variables were tested for normality using the Shapiro-Wilk test. To compare values corresponding to lymphoproliferation, expression of PD-1, T-bet, GATA3, IL-10, IFN-y, TNF-α, as well as NO_2_ production within groups, Friedman’s test with Dunn’s post-test was used. The Mann-Whitney test was used to compare results among groups. Values were considered significant when p <0.05.

## Results

### Clinical and laboratory findings in naturally infected animals

Dogs selected from the Araçatuba Zoonosis Control Center showed at least three signs compatible with CanL, including onychogryphosis and skin lesions (in 7 out of 10 dogs), lymphadenopathy (6 out of 10), periocular lesions and cachexia (5 out of 10), alopecia (4 out of 10) and ear-tip lesions (3 out of 10). Negative control animals showed no clinical manifestations (S1 Table). All 10 diseased dogs, but none of the five negative controls, presented anti-*Leishmania* antibodies (ELISA OD, mean ± standard deviation, infected dogs: 0.88 ± 0.38 vs. negative controls: 0.10 ± 0.05, cut-off value: 0.27) (S1 Table) and *Leishmania* DNA (real-time PCR, mean CT value: 27.7) (*Leishmania* DNA calibration curve CT value range: 13.2–33.7). Furthermore, the infected dogs presented statistically significant reductions in RBC counts, hematocrit, hemoglobin and serum albumin concentrations and the albumin/globulin ratio, as well as increased serum globulin concentrations, in comparison to negative controls (S2–S4 Tables). Based on clinical signs and laboratory findings, the diseased dogs showed moderate disease manifestations classified as clinical stage II leishmaniasis according to Solano-Gallego et al, 2009 [42].

In 8/10 diseased dogs, antibodies specific for *Ehrlichia spp* were detected by rapid testing. None of these dogs presented *Dirofilaria immitis* antigens or antibodies specific to *Anaplasma phagocytophilum*/*Anaplasma platys* or *Borrelia burgdorferi*. Conventional PCR carried out in blood samples failed to reveal *Ehrlichia spp* DNA in either the diseased or control dogs.

### Combinations of recombinant canine proteins, or rcasIL-10R1 alone, induced lymphoproliferation

**Fig 1:**
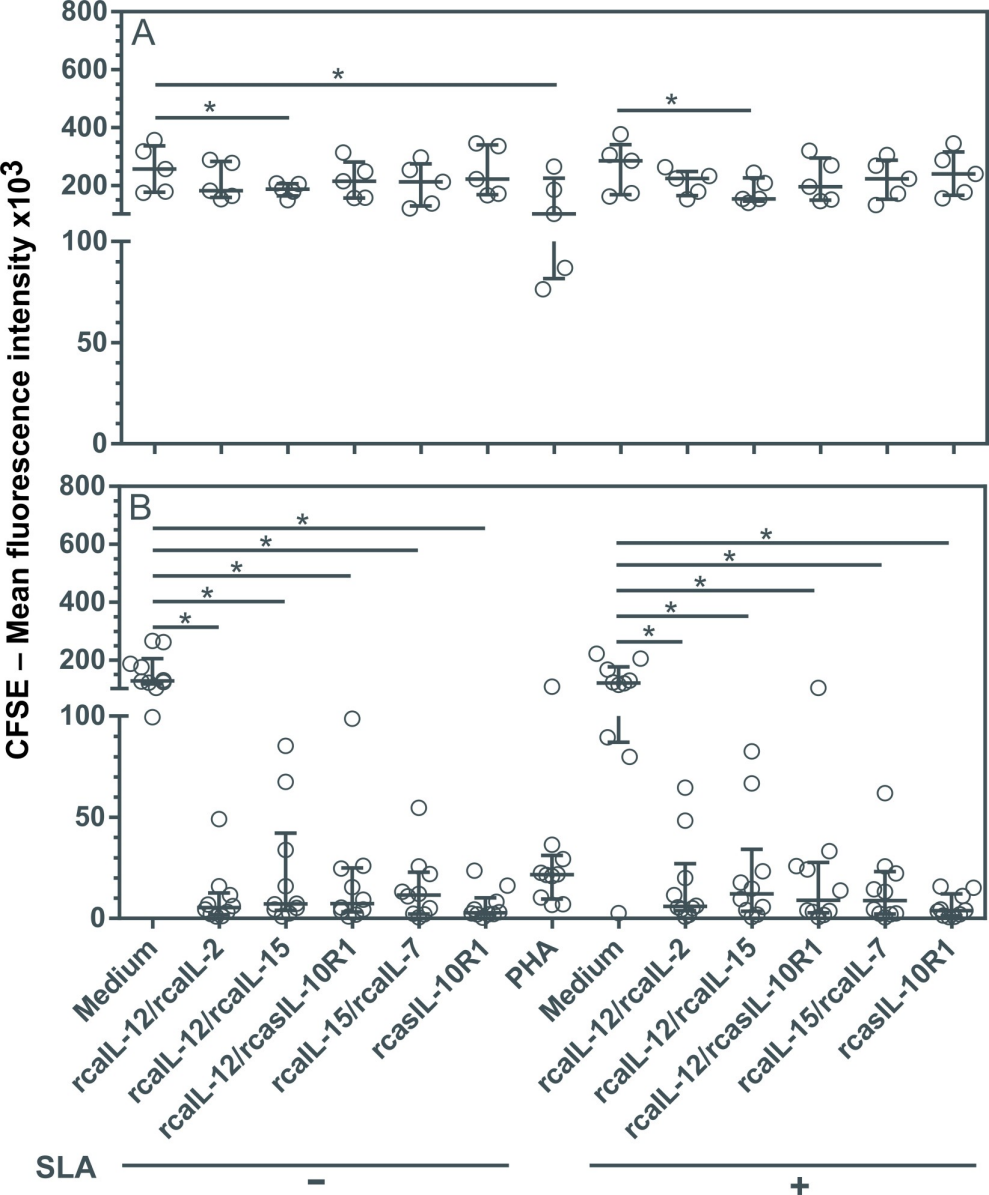
Evaluation of lymphoproliferation after stimulation with recombinant canine proteins in healthy dogs and with leishmaniasis. CFSE-labeled PBMCs from healthy negative control dogs (n = 5) (A) and dogs with leishmaniasis (n = 10) (B) were cultured in medium alone (Medium), medium with soluble *Leishmania* antigens (SLA) or phytohemagglutinin (PHA). In addition, PBMCs cultured in medium alone or with SLA were stimulated with the following recombinant canine proteins: rcaIL-12/rcaIL-2, rcaIL-12/rcaIL-15, rcaIL-12/rcasIL-10R1, rcaIL-15/rcaIL-7, or rcasIL-10R1 alone. After 5 days, the mean fluorescence intensity (MFI) of CFSE-labeled lymphocytes was assessed by flow cytometry. Bars represent MFI median values and 25^th^ and 75^th^ percentile interquartile range. Symbols represent data from individual animals. Asterisks indicate significant differences (Friedman’s test with Dunn’s multiple comparison, p < 0.05).

In dogs naturally infected with leishmaniasis, the ability to mount a lymphoproliferative response is limited after PBMCs are stimulated with *Leishmania* antigens [12,43,44]. In an attempt to develop protocols to promote a lymphoproliferative response in these dogs, combinations of rcaIL-12/rcaIL-2, rcaIL-12/rcaIL-15, rcaIL-12/rcasIL-10R1, rcaIL-15/rcaIL-7 or rcasIL-10R1 alone were tested. PBMCs from healthy or infected dogs were cultured together with, or without, the recombinant proteins, and with or without the addition of SLA, or in the presence of PHA alone for five days. The Mean Fluorescence Intensities (MFI) of CFSE-labeled lymphocytes was determined under each condition. Reductions in CFSE-fluorescence were considered an indicator of cell proliferation [39]. In healthy dogs, lymphoproliferation was observed when PBMCs were cultured with PHA or a combination of rcaIL-12/rcaIL-15, with or without the addition of SLA (Fig 1A). In *Leishmania*-infected dogs, although CFSE-labeled lymphocytes cultured with PHA showed some reductions in MFI, statistical significance was not observed (Fig 1B). Interestingly, the lymphocytes from diseased dogs exhibited a proliferative response when cultured in each of the combinations of recombinant proteins tested (rcaIL-12/rcaIL-2, rcaIL-12/rcaIL-15, rcaIL-12/rcasIL-10R1, rcaIL-15/rcaIL-7), as well as with rcasIL-10R1 alone, regardless of the addition of SLA to cultures (Fig 1B).

### Combinations of rcaIL12/rcaIL-2 and rcaIL-12/rcaIL-15 promote decreases in lymphocyte PD-1 expression

**Fig 2:**
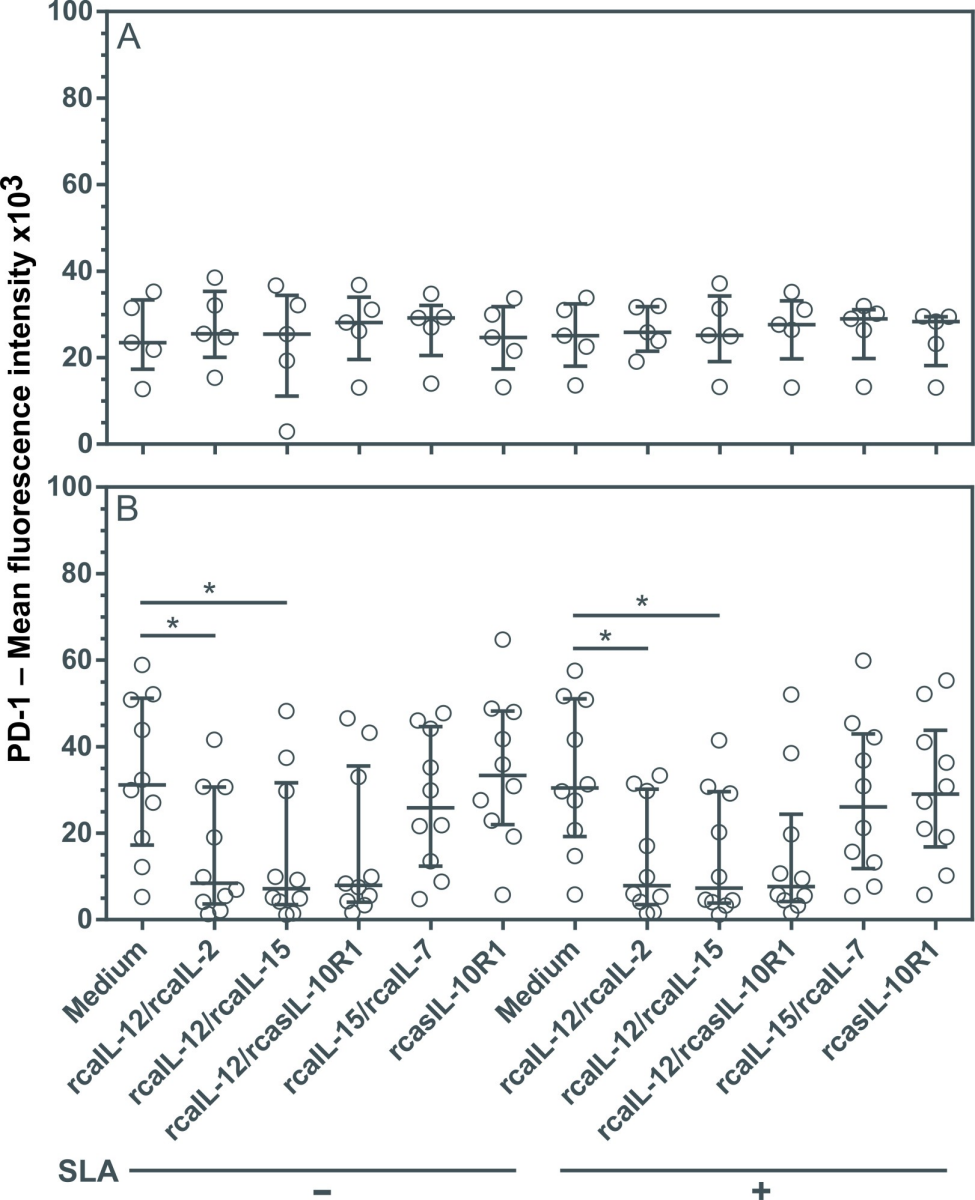
Evaluation of PD-1 expression in lymphocytes from healthy and diseased dogs after stimulation with recombinant canine proteins. PBMCs from healthy negative control dogs (n = 5) (A) and dogs with leishmaniasis (n = 10) (B) were cultured in medium alone (Medium) or medium containing rcaIL-12/rcaIL-2, rcaIL-12/rcaIL-15, rcaIL-12/rcasIL-10R1, rcaIL-15/rcaIL-7, or rcasIL-10R1 alone, with or without the addition of SLA. After 5 days, PMBCs were labeled with anti-human CD279 (PD-1) PE-conjugated monoclonal antibodies or a PE-conjugated isotype control. Lymphocyte mean fluorescence intensity (MFI) was assessed by flow cytometry. Bars represent MFI median values and 25^th^ and 75^th^ percentile interquartile range. Symbols represent data from individual animals. Asterisks indicate significant differences (Friedman’s test with Dunn’s multiple comparison, p < 0.05).

The inability of lymphocytes from dogs with leishmaniasis to proliferate and produce Th1 cytokines may be associated, at least in part, with increased PD-1 expression, which promotes apoptosis during the course of infection [18,19]. To assess whether interference in cytokine signaling could lead to reduced PD-1 expression in lymphocytes, PBMCs were cultured with or without SLA using combinations of recombinant canine proteins, or rcasIL-10R1 alone. No changes in PD-1 expression were seen in healthy dogs regardless of the addition of recombinant canine proteins, regardless of SLA stimulation (Fig 2A). However, lymphocytes from diseased dogs showed significant decreases in PD-1 expression under a combination of rcaIL-12/rcaIL-2 and rcaIL-12/rcaIL-15, both with and without SLA stimulation (Fig 2B). Although decreases in PD-1 expression were seen using rcaIL-12/rcasIL-10R1, both with and without SLA, no statistical significance was detected (Fig 2B).

### Stimulation with rcaIL-12/rcaIL-15 induces increased T-bet expression without altering levels of GATA3

Leishmaniasis progression in dogs is associated with the inability to establish an effective cellular immune response (Th1) and the mounting of an exacerbated humoral immune response (Th2) and/or the development of an immunosuppressive state [8,17,45]. The generation of Th1 or Th2 cell subsets involves the expression of master transcription factors T-bet or GATA3, respectively [46,47]. To identify the conditions capable of modifying T helper cell differentiation in dogs with leishmaniasis, PBMCs were cultured with combinations of recombinant canine proteins with or without adding SLA.

**Fig 3:**
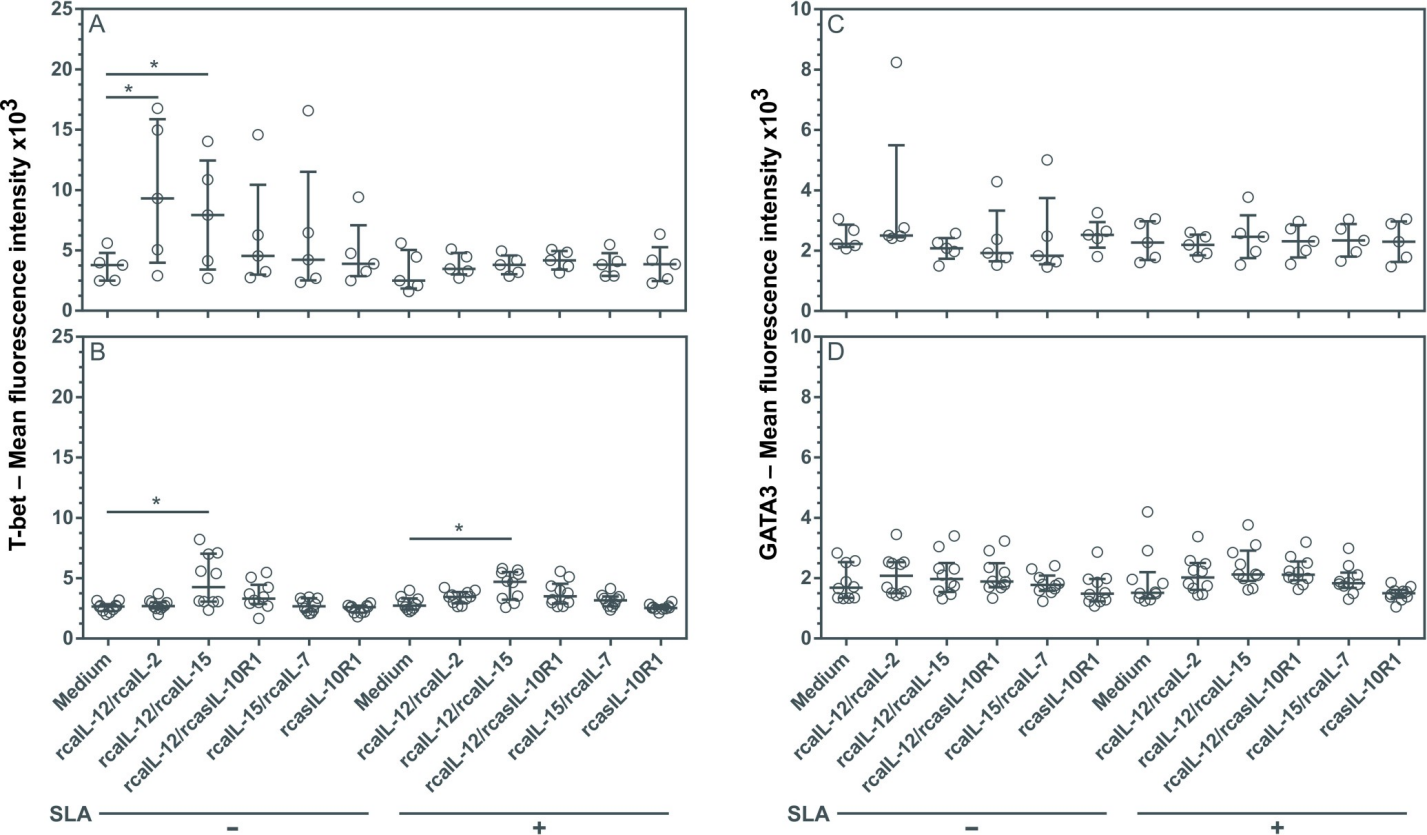
Evaluation of T-bet and GATA3 expression in lymphocytes from healthy and diseased dogs after stimulation with recombinant canine proteins. PBMCs from healthy negative control dogs (n = 5) (A, C) and dogs with leishmaniasis (n = 10) (B, D) were cultured in medium alone (Medium) or medium containing rcaIL-12/rcaIL-2, rcaIL-12/rcaIL-15, rcaIL-12/rcasIL-10R1, rcaIL-15/rcaIL-7, or rcasIL-10R1 alone, with or without SLA. After 5 days, PBMCs were labeled anti-human T-bet FITC-conjugated antibodies, and anti-human GATA3 PE-conjugated antibodies or FITC-conjugated and PE-conjugated isotype control antibodies. Lymphocyte mean fluorescence intensity (MFI) was assessed by flow cytometry. Bars represent MFI median values and 25^th^ and 75^th^ percentile interquartile range. Symbols represent data of individual animals. Asterisks indicate significant differences (Friedman’s test with Dunn’s multiple comparison, p < 0.05).

In PBMCs from healthy dogs, the combinations of rcaIL-12/rcaIL-2 or rcaIL-12/rcaIL-15, both without SLA, generated a significant increase in lymphocyte T-bet expression, which was inhibited by the addition of SLA (Fig 3A). In contrast, in PMBCs from diseased dogs, rcaIL-12/rcaIL-15 induced a significant increase in lymphocyte T-bet expression, both in the absence or presence of SLA (Fig 3B). None of the other recombinant proteins tested, either in combination or alone, were found to affect T-bet expression. In addition, none of these recombinant proteins, regardless of the presence of SLA, significantly altered the expression of GATA3 in any of the lymphocyte cultures (Fig 3C and 3D).

### Combinations of rcaIL-12/rcaIL-2, rcaIL-12/rcaIL15 and rcaIL-12/rcasIL-10R1 increased IFN-γ and TNF-α expression without altering IL-10 production

**Fig 4:**
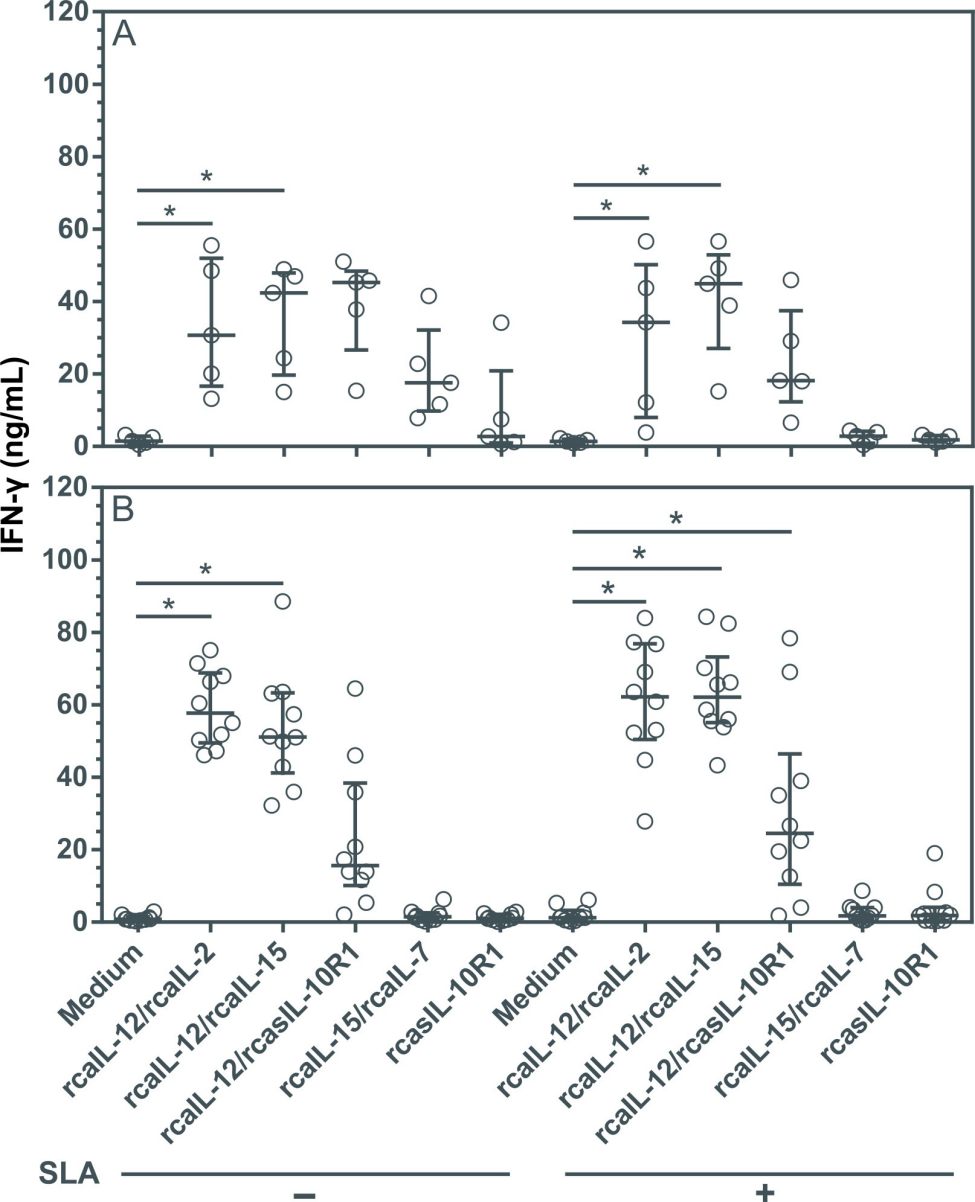
Evaluation of IFN-γ production in PBMCs from healthy and diseased dogs after stimulation with recombinant canine proteins. PBMCs from healthy negative control dogs (n = 5) (A) and dogs with leishmaniasis (n = 10) (B) were cultured in medium alone (Medium) or medium containing rcaIL-12/rcaIL-2, rcaIL-12/rcaIL-15, rcaIL-12/rcasIL-10R1, rcaIL-15/rcaIL-7, or rcasIL-10R1 alone. These PBMCs were cultured with combinations of recombinant canine proteins with or without adding SLA. After 5 days, IFN-γ concentration was determined in culture supernatants by capture ELISA. Bars represent cytokine concentration medians and 25^th^ and 75^th^ percentile interquartile range. Symbols represent data from individual animals. Asterisks indicate significant differences (Friedman’s test with Dunn’s multiple comparison, p < 0.05).

**Fig 5:**
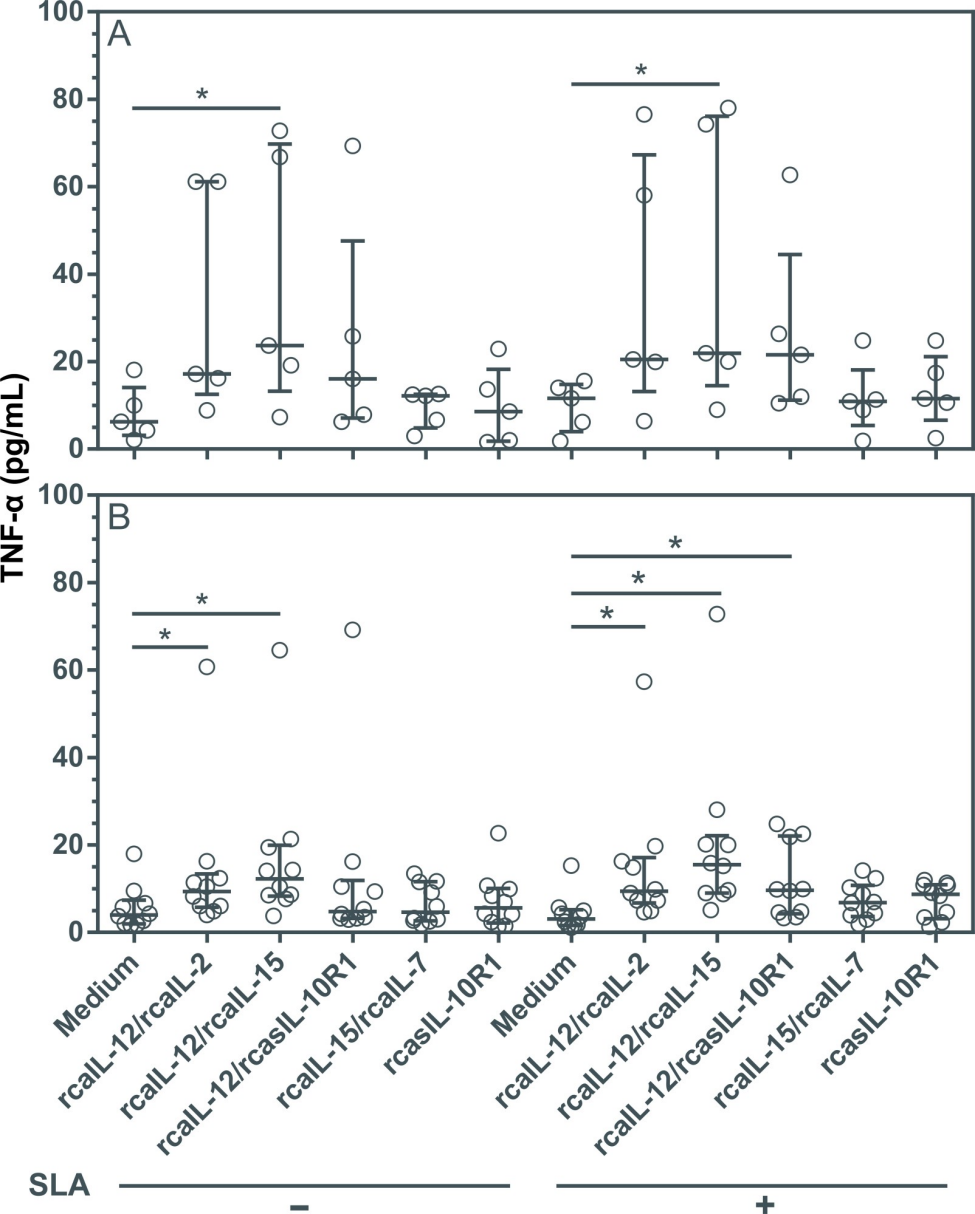
Evaluation of TNF-α production in PBMCs from healthy and diseased dogs after stimulation with recombinant canine proteins. PBMCs from healthy negative control dogs (n = 5) (A) and dogs with leishmaniasis (n = 10) (B) were cultured in medium alone (Medium) or medium containing rcaIL-12/rcaIL-2, rcaIL-12/rcaIL-15, rcaIL-12/rcasIL-10R1, rcaIL-15/rcaIL-7, or rcasIL-10R1 alone. These PBMCs were cultured with combinations of recombinant canine proteins with or without adding SLA. After 5 days, TNF-α concentration was determined in culture supernatants by capture ELISA. Bars represent cytokine concentration medians and 25^th^ and 75^th^ percentile interquartile range. Symbols represent data from individual animals. Asterisks indicate significant differences (Friedman’s test with Dunn’s multiple comparison, p < 0.05).

**Fig 6:**
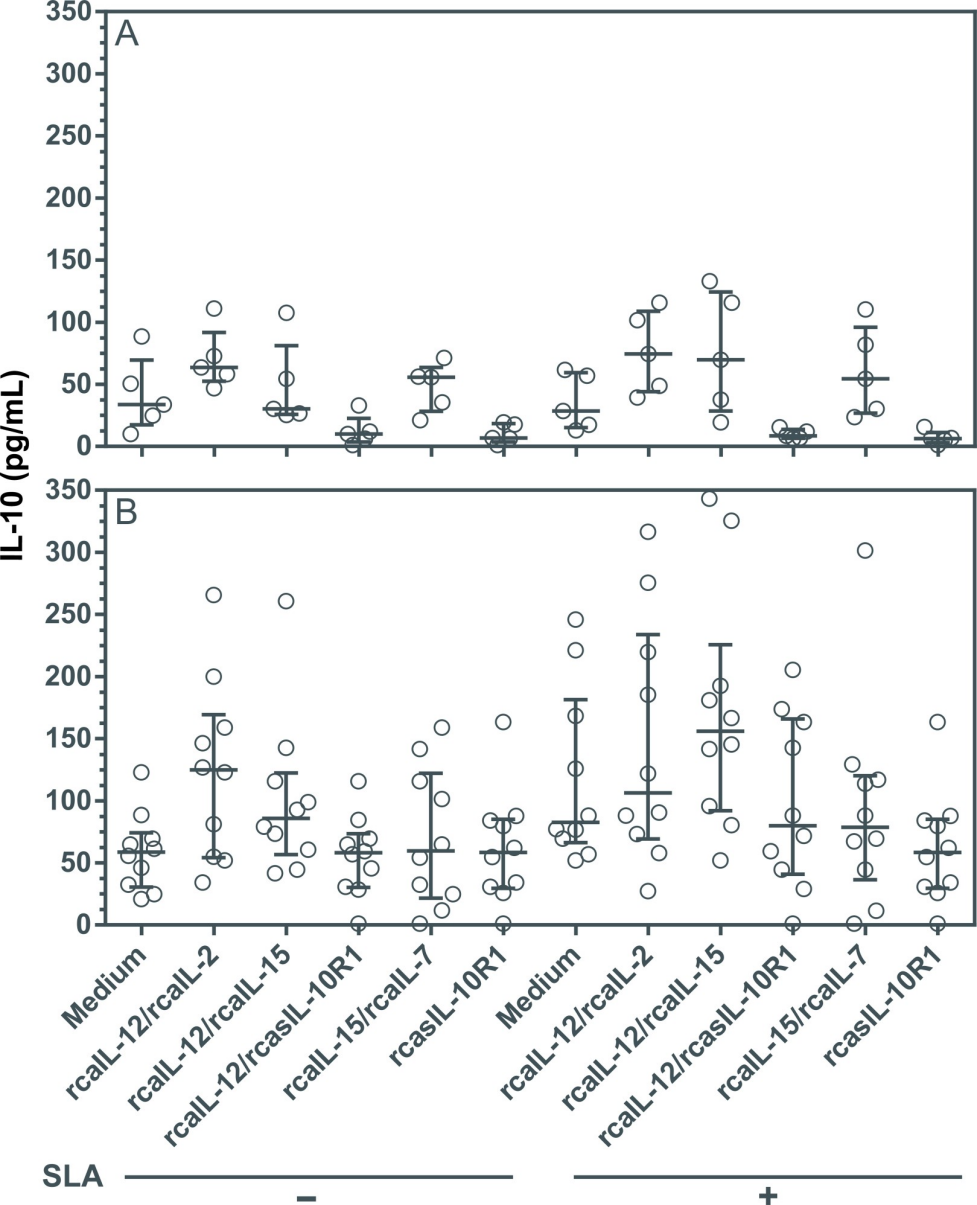
Evaluation of IL-10 production in PBMCs from healthy and diseased dogs after stimulation with recombinant canine proteins. PBMCs from healthy negative control dogs (n = 5) (A) and dogs with leishmaniasis (n = 10) (B) were cultured in medium alone (Medium) or medium containing rcaIL-12/rcaIL-2, rcaIL-12/rcaIL-15, rcaIL-12/rcasIL-10R1, rcaIL-15/rcaIL-7, or rcasIL-10R1 alone. These PBMCs were cultured with combinations of recombinant canine proteins with or without adding SLA. After 5 days, IL-10 concentration was determined in culture supernatants by capture ELISA. Bars represent cytokine concentration medians and 25^th^ and 75^th^ percentile interquartile range. Symbols represent data from individual animals. Asterisks indicate significant differences (Friedman’s test with Dunn’s multiple comparison, p < 0.05).

Driving a long-term specific Th1 immune response, while preventing Th2 and/or an immunosuppressive state, may be useful in the treatment of CanL [8,17,45]. To determine the impact on the production of cytokines mediating Th1 or immunosuppression in diseased dogs, PBMCs were cultured with combinations of recombinant proteins, or rcasIL-10R1 alone. In healthy canine PBMCs, the combinations of rcaIL-12/rcaIL-2 and rcaIL-12/rcaIL-15 induced significant increases in IFN-γ levels (Fig 4A), while the combination of rcaIL-12/rcaIL-15 induced significant increases in TNF-α levels (Fig 5A), both regardless of SLA stimulation. In PBMCs from diseased dogs, the combinations of rcaIL-12/rcaIL-2 and rcaIL-12/rcaIL-15 induced significant increases in IFN-γ and TNF-α levels (Fig 4B and Fig 5B), both in the absence or presence of SLA. In addition, in diseased dogs, the combination of rcaIL-12/rcasIL-10R1 promoted significant increases in IFN-γ and TNF-α only in the presence of SLA (Fig 4B and Fig 5B). In PBMCs from healthy and diseased dogs, no combination of recombinant proteins, regardless of the presence of SLA, significantly altered IL-10 levels (Fig 6A and 6B). Interestingly, an increasing trend in IL-10 production was noted in the cell culture supernatants of dogs with leishmaniasis, especially under SLA stimulation (Fig 6B). Finally, in PBMCs from healthy dogs, minimal IL-10 detection was observed when rcasIL-10R1 was added either alone or in combination with rcaIL-12, yet without statistical significance (Fig 6A).

### Combined rcaIL-12/rcaIL-2, or rcasIL-10R1 alone, increased NO_2_ production in the presence of SLA

**Fig 7:**
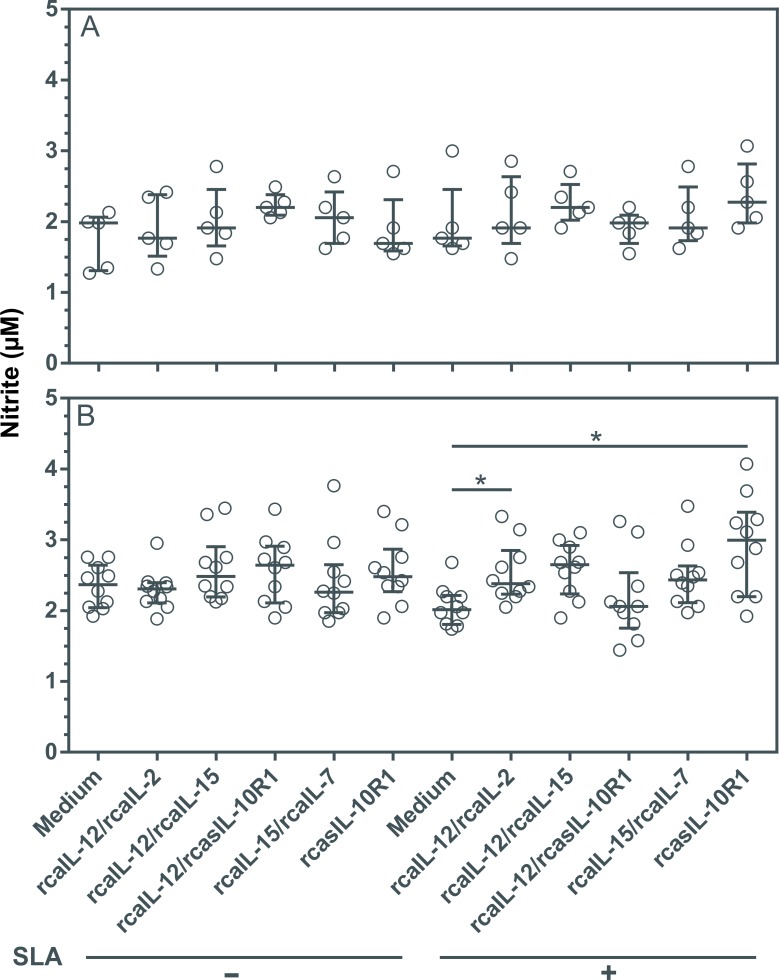
NO_2_ production in PBMCs from healthy and diseased dogs stimulated with recombinant canine proteins. PBMCs from healthy negative control dogs (n = 5) (A) and dogs with leishmaniasis (n = 10) (B) were cultured in medium alone (Medium) or medium with rcaIL-12/rcaIL-2, rcaIL-12/rcaIL-15, rcaIL-12/rcasIL-10R1, rcaIL-15/rcaIL-7, or rcasIL-10R1 alone, either in the presence or absence of SLA. After 5 days, NO_2_ concentrations was determined in culture supernatants by the Griess method. Bars represent nitrite concentration medians and 25^th^ and 75^th^ percentile interquartile range. Symbols represent data from individual animals. Asterisks indicate significant differences (Friedman’s test with Dunn’s multiple comparison, p < 0.05).

IFN-γ, TNF-α and IL-2 were associated with enhanced nitric oxide (NO) production in a canine macrophage (48). To assess whether IFN-γ and TNF-α observed in culture supernatant could lead to increased NO_2_, PBMCs were cultured with combinations of recombinant proteins, or rcasIL-10R1 alone, in the presence or absence of SLA for five days. Healthy dogs presented no changes in NO_2_ levels regardless of the addition of recombinant canine proteins and/or SLA to PMBC cultures (Fig 7A). However, significantly increased levels of NO_2_ were observed in diseased dogs when rcaIL-12/rcaIL-2 or rcaIL-10R1 was added to SLA-stimulated PBMC cultures (Fig 7B).

## Discussion

The present work considered 10 dogs presenting clinical manifestations and clinical-pathological findings compatible with CanL. *Leishmania* DNA was detected in the peripheral blood of all animals. While 8/10 of these dogs had antibodies specific for *Ehrlichia spp*. antigens, *Ehrlichia spp*. DNA was not detected in any, therefore indicating prior bacterial exposure. None of the animals presented *Dirofilaria immitis* antigens, or antibodies specific to *Anaplasma spp*. or *B*. *burgdorferi*. Together, these data provide evidence that CanL was the primary disease affecting the studied animals.

Dogs naturally infected with *Leishmania infantum* that develop the disease or present disease relapse following specific treatment, show an inability to mount a specific effective adaptive immune response, the so-called Th1 immune response with long-term memory. In order to develop immunotherapeutic protocols, this study evaluated a set of recombinant canine proteins capable of interfering with cytokine signaling pathways to determine the modulation of cellular responses in dogs with leishmaniasis.

The inability to mount an effective response in dogs with leishmaniasis occurs due to T lymphocyte exhaustion [45], involving loss of ability to perform CD4 and CD8 effector cell functions. In this study, several different combinations of recombinant proteins were shown to promote lymphoproliferation in dogs naturally infected with leishmaniasis. Lymphoproliferation occurred following incubation with rcaIL-12/rcaIL-2, rcaIL-12/rcaIL-15, rcaIL-12/rcasIL-10R1, rcaIL-15/rcaIL-7, or rcasIL-10R1 alone, regardless of whether SLA was added to cultures or not. One probable explanation for this phenomenon is the presence of *Leishmania* parasites within the PBMCs used in experimentation [49]. In fact, *Leishmania* parasites were detectable in every blood sample from each diseased dog (S1 Table).

Previously, it has been reported that stimulation with IL-12 [20] or blocking IL-10 signaling [45,50], result in restoration of specific lymphoproliferative response in dogs with leishmaniasis. The data presented here also indicate that rcaIL-12 and IL-10 blockade (by rcasIL-10R1) can contribute to the generation of lymphoproliferative response in CanL. In future experiments, the subpopulations of lymphocytes stimulated to expand by the recombinant canine proteins tested here will be determined.

None of the combinations of recombinant proteins or rcasIL-10R1 caused significant alteration in the PD1 expression of lymphocytes from the healthy negative control dogs. However, the rcaIL-12/rcaIL-2 and rcaIL-12/rcaIL-15 elicited a significant decrease in PD-1 protein expression in lymphocytes from dogs with leishmaniasis. In a previous study, stimulation with IL-12 was shown to cause a reduction in PD-1 and an increase in T-bet expression, and arouse effector function in CD8^+^ T lymphocytes, rescuing these cells from exhaustion in human patients infected with hepatitis B virus [50]. Recombinant canine IL-12 probably modulates PD-1 in part through induction in T-bet transcription factor expression [51,52].

Recombinant canine IL-12/rcaIL-2 and rcaIL-12/rcaIL-15 promoted an increase in T-bet expression in healthy negative control dog lymphocytes, which was inhibited by the addition of SLA, suggesting some suppressive SLA activity. In lymphocytes from dogs with leishmaniasis, only rcaIL-12/rcaIL-15 induced an increase in T-bet expression, independent of the addition of SLA in the cultures. Interleukin-15 may have elicited an increase in IL-12Rβ1 expression [53] resulting in a higher level of IL-12 signaling and, as a consequence, increased production T-bet. None of the recombinant canine or rcasIL-10R1 protein combinations modified the expression of GATA3 in healthy or diseased dogs.

Interestingly, dogs with leishmaniasis also showed a significant increase in IFN-γ and TNF-α production by PBMCs cultured with rcaIL-12/rcaIL-2 or rcaIL-12/rcaIL-15 combinations with or without the addition of SLA. Further, PBMCs from these animals produced significantly higher levels of IFN-γ and TNF-α after stimulation with rcaIL-12/rcasIL-10R1 and the addition of SLA in the cultures. The data showing that rcaIL-12/rcasIL-10R1 or rcasIL-10R1 have only limited effect and no effect on promoting an increase in IFN-γ production, respectively, corroborate the findings of Esch and collaborators, 2013, who reported that blockade of IL-10 signaling does not boost IFN-γ synthesis in either CD4^+^ or CD8^+^ T lymphocytes in dogs with leishmaniasis [45].

None of the combinations of recombinant proteins or rcasIL-10R1 caused significant alteration in the NO_2_ levels in culture supernatant of PBMCs from the healthy negative control dogs. However, the rcaIL-12/rcaIL-2 and rcaIL-10R1 alone elicited a significant increase in NO_2_ concentration in culture supernatant of PBMCs from dogs with leishmaniasis when SLA was added to cultures. In previous studies, stimulation with IL-2, IFN-y, TNF-α induced activation of canine macrophages and increased production of NO_2_ [48] whereas stimulation with IL-10 negatively regulated NO_2_ production in human macrophages [54,55]. In our experiments, in SLA-stimulated PMBCs from diseased dogs, the combination of rcaIL-12/rcaIL-2 and rcasIL-10R1 alone could have promoted significant increase in NO_2_ production through IFN-γ and TNF-α induction and blockade of IL-10 signaling, respectively. However, it is unclear why the combination rcasIL-12/rcaIL-15, which also promotes IFN-γ and TNF-α expression would not have stimulated a significant increase in NO_2_ synthesis.

In conclusion, among the various combinations of recombinant canine proteins and rcasIL-10R1 alone capable of interfering in the cytokine signaling pathways tested, rcaIL-12/rcaIL-15 proteins were shown to promote significant lymphoproliferative response, an increase in T-bet without altering GATA3 expression, and an increase in IFN-γ and TNF-α without changing IL-10 production. These data suggest that rcaIL-12/rcaIL-15 may enhance cellular immune responses and contribute to the reprogramming of immune responses, which is potentially useful for developing effective treatments for CanL.

## Supporting information

S1 Fig Histogram representative of flow cytometric analysis of lymphoproliferative response. CFSE-labeled PBMCs were cultured for 5 days in medium alone or medium with recombinant canine proteins. Gates R were used to delimit lymphocytes and the peaks indicated as (M) correspond to the proliferating lymphocytes. In this representative example, the data shown correspond to PBMCs from a dog with leishmaniasis cultured with medium alone (A) or medium with rcaIL-2/rcaIL-12 (B), rcaIL-12/rcaIL15 (C) rcaIL-12/rcasIL-10R1 (D), rcaIL-7/rcaIL-15 (E) or alone rcasIL-10R1 (F).
(TIF)

S2 Fig Histogram representative of the flow cytometric analysis of the labeling of PD-1. PBMCs were cultured for 5 days in medium alone or medium with rcaIL-12/rcaIL-2, rcaIL-12/rcaIL-15, rcaIL-12/rcasIL-10R1, rcaIL-15/rcaIL-7, or rcasIL-10R1 alone. Then, PMBCs were labeled with anti-human CD279 (PD-1) PE-conjugated monoclonal antibodies or PE-conjugated isotype control and lymphocyte mean fluorescence intensities (MFI) were assessed by flow cytometry. Gates R were used to delimit lymphocytes and the peaks indicated as (M) correspond to the lymphocytes expressing PD-1. In this representative example, the data shown correspond to PBMCs from a dog with leishmaniasis cultured with medium alone (A) or medium with rcaIL-2/rcaIL-12 (B), rcaIL-12/rcaIL15 (C) rcaIL-12/rcasIL-10R1 (D), rcaIL-7/rcaIL-15 (E) or alone rcasIL-10R1 (F).
(TIF)

S3 Fig Histogram representative of the flow cytometric analysis of the labeling of T-Bet and GATA3 transcription factors. PBMCs were cultured for 5 days in medium alone or medium with recombinant canine proteins. Then, PBMCs were labeled anti-human T-bet FITC-conjugated antibodies, and anti-human GATA3 PE-conjugated antibodies or FITC-conjugated and PE-conjugated isotype control antibodies, and lymphocyte mean fluorescence intensities (MFI) were assessed by flow cytometry. Gates R were used to delimit lymphocytes and the peaks indicated as (M) correspond to the lymphocytes expressing T-bet or GATA3. In this representative example, the data shown correspond to PBMCs from a dog with leishmaniasis cultured with medium alone (A) or medium with rcaIL-2/rcaIL-12 (B), rcaIL-12/rcaIL15 (C) rcaIL-12/rcasIL-10R1 (D), rcaIL-7/rcaIL-15 (E) or alone rcasIL-10R1 (F).
(TIF)

S1 Table Clinical findings, detection of anti-*Leishmania* antibodies and *Leishmania* DNA. CanL: canine leishmaniasis. Control: healthy negative control. OD: optical density. *ELISA cut-off value: OD 0.270. CT: threshold cycle. BCT: below CT value after 40 amplification cycles. **Real-time PCR calibration curve performed with DNA from 102 to 107 *Leishmania* promastigotes resulted in CT values from 13.23 to 33.74. Real-time PCR amplification specificity was confirmed by determining the melting point in each reaction.
(DOCX)

S2 Table Sera biochemical profile. CanL: canine leishmaniasis. Control: healthy negative control. ALT: alanine aminotransferase, AST: aspartate aminotransferase, GGT: gamma glutamyl transferase. a,b The same letters in the same column indicate no statistical difference using unpaired t-test.
(DOCX)

S3 Table Red blood cell parameters. CanL: canine leishmaniasis. Control: healthy negative control. RBC: red blood cells, Ht: hematocrit, MCHC: mean corpuscular hemoglobin concentration, MCV: mean corpuscular volume. a,b The same letters in the same column indicate no statistical difference using unpaired t-test.
(DOCX)

S4 Table White blood cells and platelet counts. CanL: canine leishmaniasis. Control: healthy negative control. a,b The same letters in the same column indicate no statistical difference using unpaired t-test.
(DOCX)

## References

[1] Lukes J, Mauricio IL, Schonian G, Dujardin J-C, Soteriadou K, Dedet J-P, et al Evolutionary and geographical history of the *Leishmania donovani* complex with a revision of current taxonomy. Proc Natl Acad Sci. 2007;104: 9375–9380. 10.1073/pnas.0703678104 17517634 PMC1890502

[2] Walker DM, Oghumu S, Gupta G, McGwire BS, Drew ME, Satoskar AR. Mechanisms of cellular invasion by intracellular parasites. Cell Mol Life Sci. 2014;71: 1245–1263. 10.1007/s00018-013-1491-1 24221133 PMC4107162

[3] World Health Organization. Leishmaniasis [Internet]. 2019 [cited 1 Nov 2019]. Available: https://www.who.int/news-room/fact-sheets/detail/leishmaniasis

[4] WHO. Investing to overcome the global impact of neglected tropical diseases In: World Health Organization [Internet]. 2015 [cited 21 Jan 2019]. Available: https://www.who.int/neglected_diseases/9789241564861/en/

[5] Moreno J, Alvar J. Canine leishmaniasis: epidemiological risk and the experimental model. 2002;18: 399–405. 10.1016/s1471-4922(02)02347-4 12377257

[6] Coura-Vital W, Marques MJ, Veloso VM, Roatt BM, Aguiar-Soares RD de O, Reis LES, et al prevalence and factors associated with *Leishmania infantum* infection of dogs from an urban area of Brazil as identified by molecular methods. Boelaert M, editor. PLoS Negl Trop Dis. 2011;5: e1291 10.1371/journal.pntd.0001291 21858243 PMC3156685

[7] Nunes CM, Pires MM, da Silva KM, Assis FD, Filho JG, Perri SHV. Relationship between dog culling and incidence of human visceral leishmaniasis in an endemic area. Vet Parasitol. 2010;170: 131–133. 10.1016/j.vetpar.2010.01.044 20181428

[8] Alvar J, Cañavate C, Molina R, Moreno J, Nieto J. Canine leishmaniasis. Adv Parasitol. 2004;57: 1–88. 10.1016/S0065-308X(04)57001-X 15504537

[9] Reguera RM, Morán M, Pérez-Pertejo Y, García-Estrada C, Balaña-Fouce R. Current status on prevention and treatment of canine leishmaniasis. Vet Parasitol. 2016;227: 98–114. 10.1016/j.vetpar.2016.07.011 27523945

[10] Travi BL, Cordeiro-da-Silva A, Dantas-Torres F, Miró G. Canine visceral leishmaniasis: Diagnosis and management of the reservoir living among us. PLoS Negl Trop Dis. 2018;12: 1–13. 10.1371/journal.pntd.0006082 29324838 PMC5764232

[11] Ribeiro RR, Michalick MSM, da Silva ME, dos Santos CCP, Frézard FJG, da Silva SM. Canine Leishmaniasis: An overview of the current status and strategies for control. Biomed Res Int. 2018;2018: 1–12. 10.1155/2018/3296893 29789784 PMC5896350

[12] Pinelli E, Wagenaar J, Bernadina W, Pinelli E, Killick-kendrick R, Wagenaar J, et al cellular and humoral immune responses in dogs experimentally and naturally infected with *Leishmania infantum*. Infect Immun. 1994;62: 229–235(7). 0019-9567/94/$04.00+0 8262632 10.1128/iai.62.1.229-235.1994 PMC186091

[13] Pinelli E, Gonzalo RM, Boog CJP, Rutten VPMG, Gebhard D, Del Real G, et al *Leishmania infantum*-specific T cell lines derived from asymptomatic dogs that lyse infected macrophages in a major histocompatibility complex-restricted manner. Eur J Immunol. 1995;25: 1594–1600. 10.1002/eji.1830250619 7614987

[14] Santos-Gomes GM, Rosa R, Leandro C, Cortes S, Romão P, Silveira H. Cytokine expression during the outcome of canine experimental infection by *Leishmania infantum*. Vet Immunol Immunopathol. 2002;88: 21–30. 10.1016/s0165-2427(02)00134-4 12088641

[15] Alves CF, de Amorim IFG, Moura EP, Ribeiro RR, Alves CF, Michalick MS, et al Expression of IFN-γ, TNF-α, IL-10 and TGF-β in lymph nodes associates with parasite load and clinical form of disease in dogs naturally infected with *Leishmania (Leishmania) chagasi*. Vet Immunol Immunopathol. 2009;128: 349–358. 10.1016/j.vetimm.2008.11.020 19124159

[16] Panaro MA, Acquafredda A, Lisi S, Lofrumento DD, Mitolo V, Sisto M, et al Nitric oxide production by macrophages of dogs vaccinated with killed *Leishmania infantum* promastigotes. Comp Immunol Microbiol Infect Dis. 2001;24: 187–95. Available: http://www.ncbi.nlm.nih.gov/pubmed/11440191 10.1016/s0147-9571(00)00026-6 11440191

[17] Boggiatto PM, Ramer-Tait AE, Metz K, Kramer EE, Gibson-Corley K, Mullin K, et al Immunologic indicators of clinical progression during canine Leishmania infantum infection. Clin Vaccine Immunol. 2010;17: 267–273. 10.1128/CVI.00456-09 20032217 PMC2815526

[18] Chiku VM, Silva KLO, de Almeida BFM, Venturin GL, Leal AAC, de Martini CC, et al PD-1 function in apoptosis of T lymphocytes in canine visceral leishmaniasis. Immunobiology. 2016;221: 879–888. 10.1016/j.imbio.2016.03.007 27016050

[19] Oliveira Silva KL, Marin Chiku V, Luvizotto Venturin G, Correa Leal AA, de Almeida BF, De Rezende Eugenio F, et al PD-1 and PD-L1 regulate cellular immunity in canine visceral leishmaniasis. Comp Immunol Microbiol Infect Dis. 2019;62: 76–87. 10.1016/j.cimid.2018.12.002 30711051

[20] Strauss-Ayali D, Baneth G, Shor S, Okano F, Jaffe CL. Interleukin-12 augments a Th1-type immune response manifested as lymphocyte proliferation and interferon gamma production in *Leishmania infantum*-infected dogs. Int J Parasitol. 2005;35: 63–73. 10.1016/j.ijpara.2004.10.015 15619517

[21] Carvalho EM, Bacellar O, Brownell C, Regis T, Coffman RL, Reed SG. Restoration of IFN-gamma production and lymphocyte proliferation in visceral leishmaniasis. J iImunology. 1994;152: 5949–56. Available: http://www.ncbi.nlm.nih.gov/pubmed/8207220 8207220

[22] Bacellar O, Brodskyn C, Guerreiro J, Barral-Netto M, Costa CH, Coffman RL, et al Interleukin-12 restores interferon-gamma production and cytotoxic responses in visceral leishmaniasis. J Infect Dis. 1996;173: 1515–8. 10.1093/infdis/173.6.1515 8648233

[23] D’Oliveira Júnior A, Costa SRM, Bispo Barbosa A, Orge Orge M de LG, Carvalho EM. Asymptomatic *Leishmania chagasi* infection in relatives and neighbors of patients with visceral leishmaniasis. Mem Inst Oswaldo Cruz. 1997;92: 15–20. 10.1590/S0074-02761997000100003 9302407

[24] dos Santos LR, Barrouin-Melo SM, Chang Y-F, Olsen J, McDonough SP, Quimby F, et al Recombinant single-chain canine interleukin 12 induces interferon gamma mRNA expression in peripheral blood mononuclear cells of dogs with visceral leishmaniasis. Vet Immunol Immunopathol. 2004;98: 43–48. 10.1016/j.vetimm.2003.10.006 15127840

[25] Bartee E, McFadden G. Cytokine synergy: An underappreciated contributor to innate anti-viral immunity. Cytokine. 2013;63: 237–240. 10.1016/j.cyto.2013.04.036 23693158 PMC3748162

[26] Chen X, O’Donnell MA, Luo Y. Dose-dependent synergy of Th1-stimulating cytokines on bacille Calmette-Guérin-induced interferon-γ production by human mononuclear cells. Clin Exp Immunol. 2007;149: 178–185. 10.1111/j.1365-2249.2007.03413.x 17517055 PMC1942034

[27] Marchi LHL, Paschoalin T, Travassos LR, Rodrigues EG. Gene therapy with interleukin-10 receptor and interleukin-12 induces a protective interferon-γ-dependent response against B16F10-Nex2 melanoma. Cancer Gene Ther. Nature Publishing Group; 2011;18: 110–122. 10.1038/cgt.2010.58 20885448

[28] Nielsen CM, Wolf A-S, Goodier MR, Riley EM. Synergy between common γ chain family cytokines and IL-18 potentiates innate and adaptive pathways of NK cell activation. Front Immunol. 2016;7 10.3389/fimmu.2016.00101 27047490 PMC4801862

[29] Yoshikai Y, Nishimura H. The role of interleukin 15 in mounting an immune response against microbial infections. Microbes Infect. 2000;2: 381–389. 10.1016/s1286-4579(00)00329-4 10817640

[30] Pereira AM, De Pinheiro CGM, Dos Santos LR, Teixeira NC, Chang YF, Pontes-De-Carvalho LC, et al Requirement of dual stimulation by homologous recombinant IL-2 and recombinant IL-12 for the *in vitro* production of interferon gamma by canine peripheral blood mononuclear cells. BMC Res Notes. 2014;7: 1–10. 10.1186/1756-0500-7-1 25037233 PMC4109786

[31] De Pinheiro CGM, Pedrosa MDO, Teixeira NC, Ano Bom APD, Van Oers MM, Oliveira GGDS. Optimization of canine interleukin-12 production using a baculovirus insect cell expression system Biotechnology. BMC Res Notes. BioMed Central; 2016;9: 1–11. 10.1186/s13104-016-1843-7 26795376 PMC4722752

[32] Lima VMF, Gonçalves ME, Ikeda FA, Luvizotto MCR, Feitosa MM. Anti-leishmania antibodies in cerebrospinal fluid from dogs with visceral leishmaniasis. Braz J Med Biol Res. 2003;36 Available: http://www.scielo.br/pdf/bjmbr/v36n4/4605.pdf 10.1590/s0100-879x2003000400010 12700826

[33] Perosso J, Silva KLO, Ferreira SÍ de S, Avanço SV, dos Santos PSP, Eugênio F de R, et al Alteration of sFAS and sFAS ligand expression during canine visceral leishmaniosis. Vet Parasitol. 2014;205: 417–423. 10.1016/j.vetpar.2014.09.006 25260330

[34] Labruna MB, McBride JW, Camargo LMA, Aguiar DM, Yabsley MJ, Davidson WR, et al A preliminary investigation of *Ehrlichia* species in ticks, humans, dogs, and capybaras from Brazil. Vet Parasitol. 2007;143: 189–195. 10.1016/j.vetpar.2006.08.005 16962245

[35] Dunham SP, Argyle DJ, Onions DE. The isolation and sequence of canine interleukin-2. DNA Seq. 1995;5: 177–180. 10.3109/10425179509029359 7612930

[36] Whitford M, Stewart S, Kuzio J, Faulkner P. Identification and sequence analysis of a gene encoding gp67, an abundant envelope glycoprotein of the baculovirus Autographa californica nuclear polyhedrosis virus. J Virol. 1989;63: 1393–9. Available: http://www.ncbi.nlm.nih.gov/pubmed/2644449 2644449 10.1128/jvi.63.3.1393-1399.1989 PMC247838

[37] USP (2011) Chapter, 85., Bacterial Endotoxins Test) [Internet]. Available: http://www.usp.org/harmonization-standards/pdg/general-methods/bacterial-endotoxins

[38] Tan JC, Braun S, Rong H, DiGiacomo R, Dolphin E, Baldwin S, et al Characterization of recombinant extracellular domain of human interleukin-10 receptor. J Biol Chem. 1995;270: 12906–12911. 10.1074/jbc.270.21.12906 7759550

[39] Lyons AB, Parish CR. Determination of lymphocyte division by flow cytometry. J Immunol Methods. 1994;171: 131–137. 10.1016/0022-1759(94)90236-4 8176234

[40] de Lima VMF, Fattori KR, Michelin A de F, Neto L da S, Vasconcelos R de O. Comparison between ELISA using total antigen and immunochromatography with antigen rK39 in the diagnosis of canine visceral leishmaniasis. Vet Parasitol. 2010;173: 330–333. 10.1016/j.vetpar.2010.07.012 20810216

[41] Green LC, Wagner DA, Glogowski J, Skipper PL, Wishnok JS, Tannenbaum SR. Analysis of nitrate, nitrite, and [15N]nitrate in biological fluids. Anal Biochem. 1982;126: 131–138. 10.1016/0003-2697(82)90118-x 7181105

[42] Solano-Gallego L, Koutinas A, Miró G, Cardoso L, Pennisi MG, Ferrer L, et al Directions for the diagnosis, clinical staging, treatment and prevention of canine leishmaniosis. Veterinary Parasitology. 2009 pp. 1–18. 10.1016/j.vetpar.2009.05.022 19559536

[43] Martínez-Moreno A, Moreno T, Martínez-Moreno FJ, Acosta I, Hernández S. Humoral and cell-mediated immunity in natural and experimental canine leishmaniasis. Vet Immunol Immunopathol. 1995;48: 209–220. 10.1016/0165-2427(95)05434-8 8578681

[44] Rhalem A, Sahibi H, Guessous-Idrissi N, Lasri S, Natami A, Riyad M, et al Immune response against Leishmania antigens in dogs naturally and experimentally infected with *Leishmania infantum*. Vet Parasitol. 1999;81: 173–184. Available: http://www.ncbi.nlm.nih.gov/pubmed/10190861 10.1016/s0304-4017(98)00240-4 10190861

[45] Esch KJ, Juelsgaard R, Martinez PA, Jones DE, Christine A. PD-1-mediated T cell exhaustion during visceral leishmaniasis impairs phagocyte function Kevin. J Immunol. 2013;191: 5542–5550. 10.4049/jimmunol.1301810 24154626 PMC3896087

[46] Mullen AC, High FA, Hutchins AS, Lee HW, Villarino AV, Livingston DM, et al Role of T-bet in commitment of Th1 cells before IL-12-dependent selection. Science. 2001;292: 1907–1910. 10.1126/science.1059835 11397944

[47] Pai S-Y, Truitt ML, Ho I-C. GATA-3 deficiency abrogates the development and maintenance of T helper type 2 cells. Proc Natl Acad Sci. 2004;101: 1993–1998. 10.1073/pnas.0308697100 14769923 PMC357040

[48] Pinelli E, Gebhard D, Mommaas AM, van Hoeij M, Langermans JA., Ruitenberg EJ, et al Infection of a canine macrophage cell line with *Leishmania infantum*: determination of nitric oxide production and anti-leishmanial activity. Vet Parasitol. 2000;92: 181–189. 10.1016/s0304-4017(00)00312-5 10962155

[49] Paraguai de Souza E, Esteves Pereira AP, Machado FC, Melo MF, Souto-Padrón T, Palatnik M, et al Occurrence of *Leishmania donovani* parasitemia in plasma of infected hamsters. Acta Trop. 2001;80: 69–75. Available: http://www.ncbi.nlm.nih.gov/pubmed/11495646 10.1016/s0001-706x(01)00150-4 11495646

[50] Schurich A, Pallett LJ, Lubowiecki M, Singh HD, Gill US, Kennedy PT, et al The third signal cytokine IL-12 rescues the anti-viral function of exhausted HBV-specific CD8 T cells. PLoS Pathog. 2013;9: e1003208 10.1371/journal.ppat.1003208 23516358 PMC3597507

[51] Kao C, Oestreich KJ, Paley MA, Crawford A, Angelosanto JM, Ali MA, et al Transcription factor T-bet represses expression of the inhibitory receptor PD-1 and sustains virus-specific CD8+ T cell responses during chronic infection. Nat Immunol. 2011;12: 663–671. 10.1038/ni.2046 21623380 PMC3306165

[52] Szabo SJ, Sullivan BM, Sternmann C, Satoskar AR, Sleckman BP, Glimcher LH. Distinct effects of T-bet in Th1 lineage commitment and IFN-γ production in CD4 and CD8 T cells. Science. 2002;295: 338–342. 10.1126/science.1065543 11786644

[53] Wu C, Warrier RR, Wang X, Presky DH, Gately MK. Regulation of interleukin-12 receptor β1 chain expression and interleukin-12 binding by human peripheral blood mononuclear cells. Eur J Immunol. 1997;27: 147–154. 10.1002/eji.1830270122 9022011

[54] Wu J, Cunha FQ, Liew FY, Weiser WY. IL-10 inhibits the synthesis of migration inhibitory factor and migration inhibitory factor-mediated macrophage activation. J Immunol. 1993;151: 4325–32. Available: http://www.ncbi.nlm.nih.gov/pubmed/7691945 7691945

[55] Vouldoukis I, Bécherel P-A, Riveros-Moreno V, Arock M, Da Silva O, Debré P, et al Interleukin-10 and interleukin-4 inhibit intracellular killing of Leishmania infantum and Leishmania major by human macrophages by decreasing nitric oxide generation. Eur J Immunol. 1997;27: 860–865. 10.1002/eji.1830270409 9130636

