# Supplementary material for: Combined in vitro IL-12 and IL-15 stimulation promotes cellular immune response in dogs with visceral leishmaniasis
Source: PLoS Negl Trop Dis. 2020 Jan 21;14(1):e0008021. doi: 10.1371/journal.pntd.0008021 (PMC7006941; doi:10.1371/journal.pntd.0008021)
Supplement: S1 Table — CanL: canine leishmaniasis. Control: healthy negative control. OD: optical density. *ELISA cut-off value: OD 0.270. CT: threshold cycle. BCT: below CT value after 40 amplification cycles. **Real-time PCR calibration curve performed with DNA from 102 to 107 Leishmania promastigotes resulted in CT values from 13.23 to 33.74. Real-time PCR amplification specificity was confirmed by determining the melting point in each reaction. (DOCX) [file pntd.0008021.s004.docx]

**Table S1. Clinical findings, detection of anti-*Leishmania* antibodies and *Leishmania* DNA**

| **Dog #** | **CanL** | | |  | **Control** | | |
| --- | --- | --- | --- | --- | --- | --- | --- |
|  | **Clinical**  **findings** | **ELISA OD values*** | **Real-time PCR**  **CT values**** |  | **Clinical findings** | **ELISA OD values*** | **Real-time PCR**  **CT values**** |
|  |  |  |  |  |  |  |  |
| 1 | Alopecia, lymphadenopathy, periocular lesions, onychogryphosis | 0.956 | 27.89 |  | None | 0.143 | BCT |
| 2 | Cachexia, periocular lesions, onychogryphosis, skin lesions | 0.506 | 26.38 |  | None | 0.129 | BCT |
| 3 | Cachexia, ear-tip injuries, periocular lesions, onychogryphosis | 1.131 | 32.11 |  | None | 0.055 | BCT |
| 4 | Alopecia, skin lesions, lymphadenopathy | 1.238 | 29.34 |  | None | 0.041 | BCT |
| 5 | Cachexia, onychogryphosis periocular lesions, skin lesions | 0.355 | 26.83 |  | None | 0.121 | BCT |
| 6 | Alopecia, cachexia, skin lesions, lymphadenopathy | 0.711 | 27.11 |  | - | - | - |
| 7 | Cachexia, lymphadenopathy, onychogryphosis, skin lesions | 1.315 | 24.23 |  | - | - | - |
| 8 | Ear-tip injuries, lymphadenopathy, onychogryphosis, skin lesions | 0.929 | 26.46 |  | - | - | - |
| 9 | Lymphadenopathy, onychogryphosis, skin lesions | 0.368 | 25.04 |  | - | - | - |
| 10 | Alopecia, onychogryphosis, ear-tip injuries, periocular lesions | 1.323 | 31.78 |  | - | - | - |
| **Mean±SD** | **-** | **0.883±0.379** | **27.72±2.63** |  | **-** | **0.098±0.046** | **-** |

CanL: Canine leishmaniasis. Control: healthy negative control. OD: optical density. *ELISA cut-off value: OD 0.270. CT: threshold cycle. BCT: below CT value after 40 amplification cycles. **Real-time PCR calibration curve performed with DNA from 10^2^ to 10^7^ *Leishmania* promastigotes resulted in CT values from 13.23 to 33.74. Real-time PCR amplification specificity was confirmed by determining the melting point in each reaction.
