# Supplementary material for: Combined in vitro IL-12 and IL-15 stimulation promotes cellular immune response in dogs with visceral leishmaniasis
Source: PLoS Negl Trop Dis. 2020 Jan 21;14(1):e0008021. doi: 10.1371/journal.pntd.0008021 (PMC7006941; doi:10.1371/journal.pntd.0008021)
Supplement: S2 Table — CanL: canine leishmaniasis. Control: healthy negative control. ALT: alanine aminotransferase, AST: aspartate aminotransferase, GGT: gamma glutamyl transferase. a,b The same letters in the same column indicate no statistical difference using unpaired t-test. (DOCX) [file pntd.0008021.s005.docx]

**Table S2. Sera biochemical profile.**

| **Dogs #** |  | **Albumin** | **Globulin** | **Total protein** | **Uric Acid** | **Creatinine** | **Urea** | **ALT** | **AST** | **GGT** |
| --- | --- | --- | --- | --- | --- | --- | --- | --- | --- | --- |
|  | **Reference values** | **26-33**  **g/L** | **24-44**  **g/L** | **54-71**  **g/L** | **0-2**  **mg/dl** | **0.5-1.5 mg/dL** | **1.67-8.33 mmol/L** | **21-102 UI/L** | **23-66**  **UI/L** | **1.2-6.4 UI/L** |
|  |  |  |  |  |  |  |  |  |  |  |
| CanL 1 |  | 16.4 | 68.7 | 85.1 | 0.6 | 0.9 | 3.8 | 63.0 | 63.2 | 2.3 |
| CanL 2 |  | 8.6 | 43.2 | 51.9 | 2.4 | 0.5 | 6.6 | 54.1 | 65.6 | 7.9 |
| CanL 3 |  | 15.6 | 55.2 | 70.9 | 0.5 | 0.7 | 5.3 | 84.3 | 66.7 | 1.0 |
| CanL 4 |  | 20.9 | 62.8 | 83.7 | 0.7 | 1.1 | 5.0 | 36.6 | 65.3 | 0.7 |
| CanL 5 |  | 8.8 | 65.9 | 74.7 | 0.8 | 0.7 | 7.3 | 120.6 | 116.3 | 0.7 |
| CanL 6 |  | 11.8 | 84.4 | 96.2 | 3.7 | 0.8 | 10.6 | 192.1 | 195.6 | 0.2 |
| CanL 7 |  | 13.5 | 67.2 | 80.7 | 2.0 | 1.1 | 10.0 | 81.0 | 69.3 | 0.5 |
| CanL 8 |  | 14.1 | 65.7 | 79.8 | 5.3 | 0.5 | 5.4 | 38.8 | 42.6 | 4.3 |
| CanL 9 |  | 9.9 | 32.9 | 42.8 | 2.2 | 0.7 | 3.1 | 31.5 | 40.7 | 8.6 |
| CanL 10 |  | 12.4 | 62.6 | 75.0 | 1.7 | 0.6 | 2.7 | 24.1 | 44.2 | 4.2 |
| **Mean±SD** | | **13.2±3.8^a^** | **60.9±14.3^a^** | **74.1±15.9^a^** | **2.0±1.5^a^** | **0.8±0.2^a^** | **6.0±2.7^a^** | **72.6±51.3^a^** | **77.0±46.9^a^** | **3.0±3.1^a^** |
| Control 1 |  | 29.3 | 30.6 | 60.0 | 2.0 | 1.4 | 9.2 | 77.4 | 46.0 | 2.5 |
| Control 2 |  | 33.7 | 29.3 | 63.0 | 1.5 | 1.1 | 6.0 | 47.8 | 45.3 | 4.5 |
| Control 3 |  | 29.1 | 23.9 | 53.0 | 0.9 | 1.1 | 4.2 | 52.3 | 29.1 | 2.4 |
| Control 4 |  | 21.6 | 39.4 | 61.1 | 1.6 | 1.0 | 5.2 | 63.5 | 58.1 | 2.5 |
| Control 5 |  | 30.1 | 30.6 | 60.7 | 1.6 | 1.3 | 7.6 | 20.1 | 43.1 | 2.8 |
| **Mean±SD** | | **28.8±4.4^b^** | **30.8±5.6^b^** | **59.6±3.8^b^** | **1.5±0.4^a^** | **1.2±0.2^a^** | **6.4±2.0^a^** | **52.2±21.3^a^** | **44.3±10.3^a^** | **2.9±0.9^a^** |

CanL: Canine leishmaniasis. Control: healthy negative control. ALT: alanine aminotransferase, AST: aspartate aminotransferase, GGT: gamma glutamyl transferase. Unpaired t-tests were carried out: ^a^ and ^a,b^ in the same column indicates no statistical difference and significant statistical difference, respectively.
