# Supplementary material for: Combined in vitro IL-12 and IL-15 stimulation promotes cellular immune response in dogs with visceral leishmaniasis
Source: PLoS Negl Trop Dis. 2020 Jan 21;14(1):e0008021. doi: 10.1371/journal.pntd.0008021 (PMC7006941; doi:10.1371/journal.pntd.0008021)
Supplement: S3 Table — CanL: canine leishmaniasis. Control: healthy negative control. RBC: red blood cells, Ht: hematocrit, MCHC: mean corpuscular hemoglobin concentration, MCV: mean corpuscular volume. a,b The same letters in the same column indicate no statistical difference using unpaired t-test. (DOCX) [file pntd.0008021.s006.docx]

**Table S3. Red blood cell parameters.**

| **Dog #** |  | **RBC** | **Hematocrit** | **Hemoglobin** | **MCV** | **MCHC** |
| --- | --- | --- | --- | --- | --- | --- |
|  | **Reference values** | **5.5-8.5 x10^6^/µL** | **37-55%** | **12-18**  **g/dL** | **60-77**  **fL** | **32-36%** |
| CanL 1 |  | 3.75 | 24.0 | 7.5 | 64.2 | 31.2 |
| CanL 2 |  | 4.08 | 24.5 | 7.7 | 60.1 | 31.4 |
| CanL 3 |  | 3.20 | 24.7 | 7.1 | 77.2 | 28.7 |
| CanL 4 |  | 5.94 | 38.5 | 12.6 | 64.9 | 32.7 |
| CanL 5 |  | 5.01 | 35.0 | 11.8 | 69.9 | 33.7 |
| CanL 6 |  | 2.49 | 16.7 | 5.2 | 67.1 | 31.1 |
| CanL 7 |  | 2.37 | 18.5 | 6.0 | 78.1 | 32.4 |
| CanL 8 |  | 3.74 | 17.0 | 8.5 | 64.2 | 35.4 |
| CanL 9 |  | 3.62 | 19.9 | 7.2 | 55.1 | 36.1 |
| CanL 10 |  | 4.30 | 28.8 | 10.0 | 67.0 | 34.7 |
| **Mean±SD** | | **3.85±1.08^a^** | **24.8±7.4^a^** | **8.4±2.4^a^** | **66.8±7.0^a^** | **32.7±2.3^a^** |
|  |  |  |  |  |  |  |
| Control 1 |  | 8.26 | 55.0 | 18.1 | 66.5 | 32.7 |
| Control 2 |  | 7.21 | 53.8 | 18.8 | 74.7 | 34.9 |
| Control 3 |  | 5.32 | 41.2 | 12.9 | 77.5 | 31.3 |
| Control 4 |  | 7.43 | 52.7 | 15.4 | 76.4 | 35.9 |
| Control 5 |  | 7.96 | 53.9 | 16.8 | 75.3 | 34.7 |
| **Mean±SD** | | **7.24±1.15^b^** | **51.3±5.7^b^** | **16.4±2.3^b^** | **74.1±4.4^a^** | **33.9±1.9^a^** |

CanL: Canine leishmaniasis. Control: healthy negative control. RBC: red blood cells, MCV: mean corpuscular, MCHC: mean corpuscular hemoglobin concentration volume. Unpaired t-tests were carried out: ^a^ and ^a,b^ in the same column indicates no statistical difference and significant statistical difference, respectively.
