# Supplementary material for: Combined in vitro IL-12 and IL-15 stimulation promotes cellular immune response in dogs with visceral leishmaniasis
Source: PLoS Negl Trop Dis. 2020 Jan 21;14(1):e0008021. doi: 10.1371/journal.pntd.0008021 (PMC7006941; doi:10.1371/journal.pntd.0008021)
Supplement: S4 Table — CanL: canine leishmaniasis. Control: healthy negative control. a,b The same letters in the same column indicate no statistical difference using unpaired t-test. (DOCX) [file pntd.0008021.s007.docx]

**Table S4. White blood cells and platelet counts.**

| **Dog #** |  | **Leukocytes** | **Neutrophils** | **Lymphocytes** | **Monocytes** | **Eosinophils** | **Basophils** | **Platelets** |
| --- | --- | --- | --- | --- | --- | --- | --- | --- |
|  | **Reference values** | **6-17 x10³/µL** | **3,000-11,000/µL** | **1,000- 4,800/µL** | **150- 1,350/µL** | **150- 1,250/µL** | **Rares/µL** | **160-430 x10³/µL** |
|  |  |  |  |  |  |  |  |  |
| CanL 1 |  | 9.8 | 6,272 | 2156 | 392 | 980 | 0 | 145 |
| CanL 2 |  | 23.9 | 13,076 | 2760 | 478 | 478 | 0 | 165 |
| CanL 3 |  | 10.2 | 7,752 | 1836 | 510 | 102 | 0 | 82 |
| CanL 4 |  | 6.3 | 4,158 | 1071 | 756 | 63 | 0 | 159 |
| CanL 5 |  | 12.5 | 9,500 | 1375 | 1,125 | 500 | 0 | 420 |
| CanL 6 |  | 14.9 | 9,238 | 3427 | 1,043 | 1,192 | 0 | 277 |
| CanL 7 |  | 12.5 | 10,000 | 1750 | 375 | 375 | 0 | 331 |
| CanL 8 |  | 14.8 | 6,808 | 5032 | 2,220 | 740 | 0 | 168 |
| CanL 9 |  | 8.7 | 6,264 | 2001 | 174 | 261 | 0 | 95 |
| CanL 10 |  | 11.8 | 10,738 | 708 | 354 | 200 | 0 | 60 |
| **Mean±SD** | | **12.5±4.8^a^** | **8381±2621^a^** | **2212±1265^a^** | **743±603^a^** | **489±377^a^** | **0±0** | **190±116^a^** |
|  |  |  |  |  |  |  |  |  |
| Control 1 |  | 10.2 | 6,324 | 3468 | 102 | 306 | 0 | 341 |
| Control 2 |  | 8.8 | 6,160 | 1408 | 880 | 352 | 0 | 296 |
| Control 3 |  | 13.1 | 6,419 | 6668 | 174 | 261 | 0 | 341 |
| Control 4 |  | 8.4 | 4,480 | 1024 | 512 | 384 | 0 | 211 |
| Control 5 |  | 10.6 | 7,844 | 1060 | 424 | 1,250 | 0 | 228 |
| **Mean±SD** | | **10.2±1.9^a^** | **6,245±1,195^a^** | **2726±2424^a^** | **418±309^a^** | **261±152^a^** | **0±0** | **283±61^a^** |

CanL: Canine leishmaniasis. Control: healthy negative control. Unpaired t-tests were carried out: ^a^ and ^a,b^ in the same column indicates no statistical difference and significant statistical difference, respectively.
